# Photobiomodulation of Cytochrome c Oxidase by Chronic Transcranial Laser in Young and Aged Brains

**DOI:** 10.3389/fnins.2022.818005

**Published:** 2022-03-18

**Authors:** Fabrízio dos Santos Cardoso, Douglas W. Barrett, Zachary Wade, Sérgio Gomes da Silva, F. Gonzalez-Lima

**Affiliations:** ^1^Department of Psychology, Institute for Neuroscience, The University of Texas at Austin, Austin, TX, United States; ^2^Núcleo de Pesquisas Tecnológicas, Universidade de Mogi das Cruzes, Mogi das Cruzes, Brazil; ^3^Departamento de Farmacologia, Faculdade de Medicina de Ribeirão Preto, Universidade de São Paulo, Ribeirão Preto, Brazil; ^4^Centro Universitário UNIFAMINAS, Muriaé, Brazil; ^5^Hospital do Câncer de Muriaé, Fundação Cristiano Varella, Muriaé, Brazil

**Keywords:** brain photobiomodulation, cytochrome oxidase histochemistry, aging, chronic laser therapy, controlled study

## Abstract

In cellular bioenergetics, cytochrome c oxidase (CCO) is the enzyme responsible for oxygen consumption in the mitochondrial electron transport chain, which drives oxidative phosphorylation for adenosine triphosphate (ATP) production. CCO is also the major intracellular acceptor of photons in the light wavelengths used for photobiomodulation (PBM). Brain function is critically dependent on oxygen consumption by CCO for ATP production. Therefore, our objectives were (1) to conduct the first detailed brain mapping study of the effects of PBM on regional CCO activity, and (2) to compare the chronic effects of PBM on young and aged brains. Specifically, we used quantitative CCO histochemistry to map the differences in CCO activity of brain regions in healthy young (4 months old) and aged (20 months old) rats from control groups with sham stimulation and from treated groups with 58 consecutive days of transcranial laser PBM (810 nm wavelength and 100 mW power). We found that aging predominantly decreased regional brain CCO activity and systems-level functional connectivity, while the chronic laser stimulation predominantly reversed these age-related effects. We concluded that chronic PBM modified the effects of aging by causing the CCO activity on brain regions in laser-treated aged rats to reach levels similar to those found in young rats. Given the crucial role of CCO in bioenergetics, PBM may be used to augment brain and behavioral functions of older individuals by improving oxidative energy metabolism.

## Introduction

Cytochrome c oxidase (CCO) is the mitochondrial enzyme responsible for reducing oxygen to water in the electron transport chain, thereby promoting cellular bioenergetics due to increased oxidative phosphorylation for adenosine triphosphate (ATP) production (Hatefi, 1985). CCO is also the major intracellular acceptor of photons in the red to near-infrared wavelengths used for photobiomodulation (PBM) (Karu, 1999). Red to near-infrared light is absorbed by the chromophores in CCO and promotes changes in the redox state of enzymes in the mitochondrial inner membrane, which promote an improvement in energy metabolism due to the increased ATP synthesis by mitochondria (Karu et al., 1995), and release of mitochondrial reactive oxygen species and nitric oxide (Karu et al., 2005). The greater availability of ATP allows the activation of kinases that induce the release of calcium and the formation of cyclic adenosine monophosphate (cAMP), which act as second messengers and activate metabolic pathways at the nuclear level (Rojas and Gonzalez-Lima, 2011; Cardoso et al., 2021b). Therefore, CCO is the primary molecular target mediating the multiple effects of PBM on neurons (Wong-Riley et al., 2005). Since neuronal activity is highly dependent on CCO activity (Wong-Riley, 1989), PBM provides a novel approach to modulate bioenergetics in the brain. Recent studies showed that transcranial PBM can modulate CCO and improve oxygenation of the prefrontal cortex of rats (Rojas et al., 2012) and humans (Tian et al., 2016; Wang et al., 2017a; Holmes et al., 2019; Pruitt et al., 2020; Saucedo et al., 2021) and this bioenergetic action is not due to a thermal effect (Wang et al., 2017b). However, there are no detailed brain mapping studies of the effects of chronic PBM on CCO activity in animals or humans.

In particular, transcranial PBM (using the same laser parameters investigated in the present study) triggers a cascade of intracellular events, including modulation of mitochondrial function, metabolite production and signaling pathways linked to cell survival and bioenergetics (Cardoso et al., 2021a,b). Depending on the cellular environment, these cellular changes can be adaptive and promote the improvement of neuronal physiology that translates into neurological and psychological improvement (Rojas and Gonzalez-Lima, 2013). During brain aging, aerobic metabolism is impaired along with alterations in CCO activity (Cardoso et al., 2021c). Recently, beneficial effects of PBM have been documented in the aging brain (Salgado et al., 2015; Salehpour et al., 2017, 2018; Vargas et al., 2017; Cardoso et al., 2021c,d; O’Donnell et al., 2021; Saucedo et al., 2021). For instance, Vargas et al. (2017) conducted a study exposing healthy elderly people to laser treatment and observed an improvement in cognitive functions in the psychomotor vigilance task, a test of sustained attention, and the delayed match-to-sample, a test of visual working memory. In D-Galactose-induced aging mice, laser PBM was able to improve cognitive performance in the Barnes and Lashley III maze and the What-Where-Which occasion task (Salehpour et al., 2017). These authors observed that memory improvement was accompanied by increased levels of ATP and improved mitochondrial function.

Other studies have demonstrated similar effects of PBM on CCO activity in excitable cells in the retina, muscle and brain (Rojas et al., 2008, 2012; Hayworth et al., 2010; Wang et al., 2016, 2017a). For instance, Hayworth et al. (2010) observed that PBM increases CCO activity in rat skeletal muscle, and Wang et al. (2016) found similar effects in the human forearm. PBM using lasers directed at the forehead induces up-regulation of CCO and hemodynamics in the human prefrontal cortex (Wang et al., 2017a), with different effects in younger and older adults (Saucedo et al., 2021).

Five recent human controlled studies showed that transcranial laser PBM resulted in acute improvements in cognitive functions such as attention and working memory (Barrett and Gonzalez-Lima, 2013; Hwang et al., 2016; Holmes et al., 2019), executive function (Blanco et al., 2015), and rule-based category learning (Blanco et al., 2017). One controlled study has reported that repeated transcranial laser PBM treatments (Vargas et al., 2017) resulted in improvements in cognitive measures of attention and working memory. However, there are no controlled studies with detailed comparisons of the cognitive improvements observed in older adults based on the functional connectivity effects of PBM. While there are some promising case reports, additional controlled studies are needed to evaluate the cognitive effects of chronic daily PBM treatments in humans, and how they relate to changes in functional connectivity.

In view of these findings, this study aimed to map the differences in CCO activity of brain regions of healthy young and aged rats submitted to laser PBM for 58 consecutive days. Our guiding hypothesis was that chronic PBM would affect regional CCO activity in the brain, but that PBM-induced brain effects would be different for young and aged rats.

## Materials and Methods

### Animals

Twenty-four male Wistar rats, young (4 months old) and aged (20 months old), were used in this study. The animals were housed at a temperature of 21 ± 2°C with a 12 h light/dark cycle (lights on from 7 am to 7 pm), and food and water were provided *ad libitum* throughout the experimental period. All procedures were approved by the ethics committee of the University of Mogi das Cruzes (UMC) (# 004/2020) and all effort was made to minimize animal suffering in accordance with the proposals of the International Ethical Guidelines for Biomedical Research (Council for International Organizations of Medical Sciences [CIOMS], 1985). The 24 frozen brains were processed and analyzed at the University of Texas at Austin following all the institutional laboratory safety guidelines.

### Laser Treatment

**FIGURE 1:**
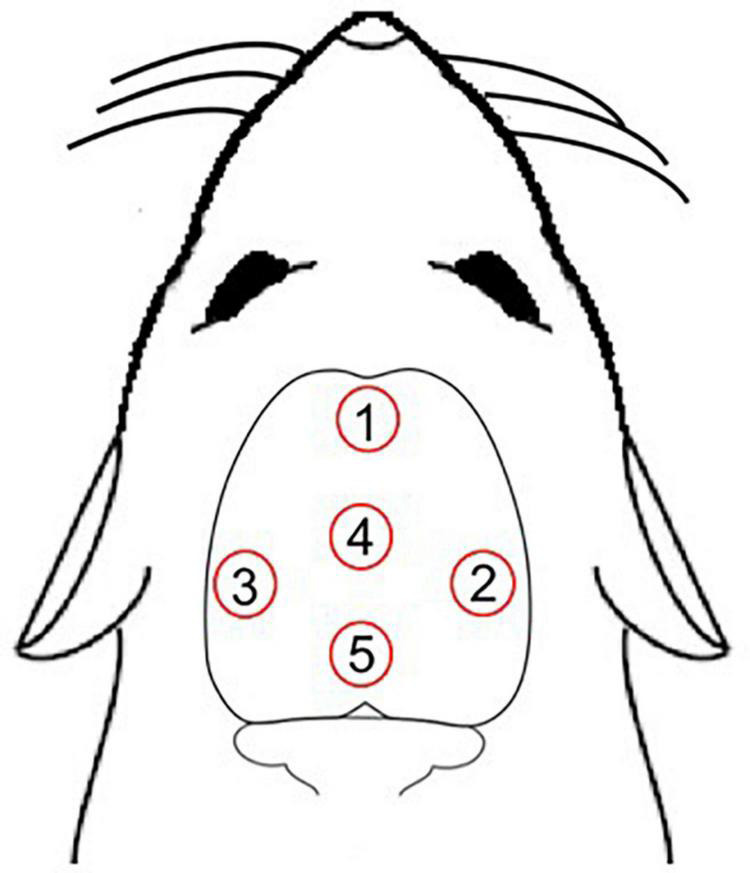
Five irradiation points over the scalp. Point 1, targeted frontal lobe; point 2, targeted right posterior parietal and temporal lobes; point 3, targeted left posterior parietal and temporal lobes; point 4, targeted anterior parietal lobe; point 5, targeted occipital lobe (Paxinos and Watson, 2006).

The rats were randomly distributed into four groups: young laser (YL; *n* = 5), young control (YC; *n* = 7), aged laser (AL; *n* = 6) and aged control (AC; *n* = 6). One week before the treatment protocol, the animals were adapted daily to the manual handling used for immobilization, which served to minimize any discomfort of the animals during the protocol. After this, rats from laser groups (YL and AL) were manually immobilized (without anesthesia) and received the treatment with a laser diode of 810 nm wavelength and 100 mW power for 30 s at each point of application. Animals from control groups (YC and AC) received the same procedure as the laser groups, but as placebo/sham treatment (laser off). We used five irradiation points on top of the skullcap (Figure 1), which were chosen to target most of the brain. Their approximate antero-posterior (AP) and medial-lateral (ML) atlas coordinates and corresponding brain targets were: point 1 = AP + 4.20 mm, ML 0.00 mm, targeted frontal lobe; point 2 = AP −3.00 mm, ML −6.60 mm, targeted right posterior parietal and temporal lobes; point 3 = AP −3.00 mm, ML + 6.60 mm, targeted left posterior parietal and temporal lobes; point 4 = AP 0.00 mm, ML 0.00 mm, targeted anterior parietal lobe; point 5 = AP −5.52 mm, ML 0.00 mm, targeted occipital lobe (Paxinos and Watson, 2006). Thus, the main targeted brain regions were part of the following functional neuroanatomical systems: sensorimotor (frontoparietal cortex, posterior thalamus, and caudate-putamen), limbic (hippocampus and anterior thalamus), auditory (temporal cortex and medial geniculate thalamus), and visual (occipital cortex and lateral posterior thalamus). The total daily laser treatment was 15 Joules of energy, 150 s of irradiation and fluence of 609 J/cm^2^. No difference in scalp temperature measured in the animals was observed with a non-contact thermometer during the treatment protocol. Animals were exposed to the transcranial laser for 58 consecutive days. This long-time frame was used because animal experimentation allowed us to focus on the effects of chronic treatment, instead of the acute effects we have studied in humans. The chosen laser parameters listed below were based on previous publications, which indicated that these parameters had anti-inflammatory properties and metabolic effects in different animal models (Almeida et al., 2013; Haslerud et al., 2017; Tomazoni et al., 2017; Naterstad et al., 2018; Cardoso et al., 2021a,b,d).

List of laser parameters:

Center wavelength (nm): 810.

Operating mode: Continuous wave (CW).

Average radiant power (W): 0.10.

Aperture diameter (cm): 0.177.

Irradiance at aperture (W/cm^2^): 4.06.

Beam divergence: 21.71°.

Beam shape: Circular.

Beam spot size (cm^2^): 0.0246.

Exposure duration/point (s): 30.

Radiant exposure (J/cm^2^) per point per session: 121.8.

Number of points irradiated: Five.

Delivery mode: Contact mode.

Number and frequency of sessions: one session/day for 58 consecutive days.

Total radiant energy (J) per head: 15.

### Quantitative Histochemistry of Brain Cytochrome Oxidase

Quantitative CCO histochemical activity data reveals the capacity for the holoenzyme to change its catalytic activity over a period of time to give a historical account of tissue changes in metabolic oxidative energy (Gonzalez-Lima and Cada, 1998a). After the laser sessions, the brains of rats were immediately removed and frozen in isopentane. Coronal sections of the brain (40 μm thick) were obtained using a cryostat (Reichert-Jung) at −20°C and processed for CCO histochemistry, following the quantitative method previously described in detail by Gonzalez-Lima and Cada (1998a).

Briefly, calibration slides were prepared with cryostat sections of different thicknesses (10, 20, 40, 60, and 80 μm) made from frozen brain paste homogenate of known CCO activity measured by spectrophotometry. Slides with sets of the sections from the brain paste homogenate were used as calibration standards in each CCO staining bath. Series of coronal sections from each brain, along with a set of paste homogenate standards, were used for the CCO histochemistry. In summary, the slides were fixed for 5 min with 0.5% vol/vol glutaraldehyde and rinsed three times in 0.1 M phosphate buffer with 10% wt/vol sucrose (pH 7.6). Then, the slides were pre-incubated in a solution containing 275 mg/l cobalt chloride, 10% wt/vol sucrose and 0.5% vol/vol dimethyl sulfoxide dissolved in Tris buffer (pH 7.6). After this, the slides were incubated at 37°C for 1 h in the dark with continuous stirring in a solution containing 350 mg diaminobenzidine tetrahydrochloride, 35 g sucrose, 52.5 mg cytochrome c and 14 mg catalase dissolved in 700 ml of oxygen-saturated 0.1 M phosphate buffer (pH 7.6). Finally, the slides were dehydrated in ethanol baths (increasing from 30 to 100% vol/vol ethanol), cleared with xylene, and cover slipped with Permount.

**FIGURE 2:**
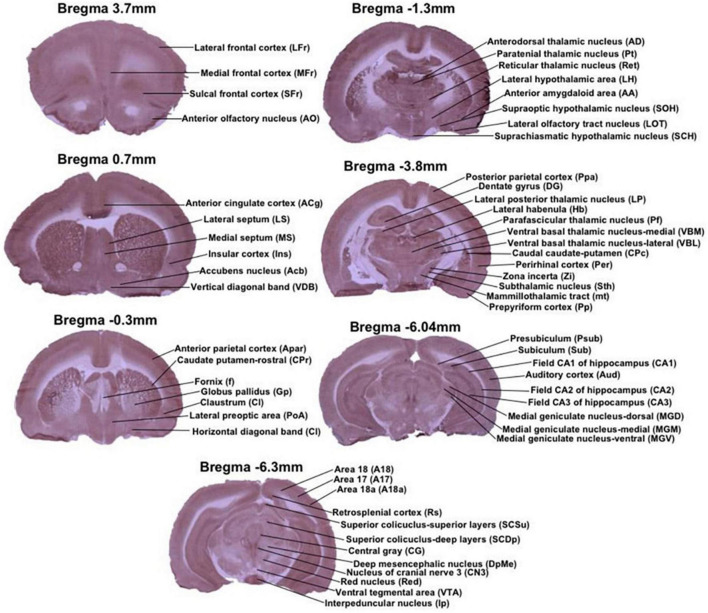
Regions of interest from representative stained sections used in this study. Each image is a coronal brain section histochemically stained for cytochrome oxidase activity and depicting regions of interest by Bregma level. Anterior–posterior Bregma coordinates are indicated on top of each image. All of the stained sections in figure are CCO-stained sections, that are presented as exemplars of the CCO-stained sections analyzed in this study.

Cytochrome c oxidase histochemical staining intensity was measured by densitometric analysis using a computer-assisted image analysis workstation consisting of a high-precision illuminator and digital camera, and a computer with image analysis software (ImageJ). Optical density (OD) readings were obtained from the following brain regions of interest chosen using the representative sample from the CCO atlas of Gonzalez-Lima and Cada (1998b): anterior olfactory nucleus (AO), lateral frontal cortex (LFr), medial frontal cortex (MFr) and sulcal frontal cortex (SFr) from Bregma 3.7 mm; accumbens nucleus (Acb), anterior cingulate cortex (ACg), insular cortex (Ins), lateral septum (LS), medial septum (MS) and vertical diagonal band (VDB) from Bregma 0.7 mm; anterior parietal cortex (APar), caudate putamen-rostral (CPr), claustrum (Cl), fornix (f), globus pallidus (Gp), horizontal diagonal band (HDB), and lateral preoptic area (PoA) from Bregma −0.3 mm; anterior amygdaloid area (AA), anterodorsal thalamic nucleus (AD), lateral hypothalamic area (LH), lateral olfactory tract nucleus (LOT), paratenial thalamic nucleus (Pt), reticular thalamic nucleus (Ret), suprachiasmatic hypothalamic nucleus (SCH), and supraoptic hypothalamic nucleus (SOH) from Bregma −1.3 mm; caudal caudate-putamen (CPc), dentate gyrus (DG), lateral habenula (Hb), lateral posterior thalamic nucleus (LP), mammillothalamic tract (mt), perirhinal cortex (Per), parafascicular thalamic nucleus (Pf), prepyriform cortex (Pp), posterior parietal cortex (Ppa), subthalamic nucleus (Sth), ventral basal thalamic nucleus-lateral (VBL), ventral basal thalamic nucleus-medial (VBM), and zona incerta (Zi) from Bregma −3.8; auditory cortex (Aud), field CA1 of hippocampus (CA1), field CA2 of hippocampus (CA2), field CA3 of hippocampus (CA3), medial geniculate nucleus-dorsal (MGD), medial geniculate nucleus-medial (MGM), medial geniculate nucleus-ventral (MGV), presubiculum (Psub), subiculum (Sub) from Bregma −6.04 mm; Area 17 (A17), Area 18 (A18), Area 18a (A18a), central gray (CG), nucleus of cranial nerve 3 (CN3), deep mesencephalic nucleus (DpMe), interpeduncular nucleus (Ip), retrosplenial cortex (Rs), red nucleus (Red), superior colliculus-deep layers (SCDp), superior colliculus-superior layers (SCSu), and ventral tegmental area (VTA) from Bregma −6.3 mm (Figure 2).

### Statistical Analyses

First, we calculated descriptive statistics to be able to establish rigorous group comparisons. To calibrate for possible variations in staining between brain sections of different staining baths, measurements were made from the CCO-stained brain standards. For each incubation bath, regression curves were calculated between the section thickness and the known CCO activity level in each set of standards. Mean OD measured in each brain region was converted into CCO activity units (μmol of cytochrome c oxidized/min/g of wet tissue weight) using the regression curve calculated in each standard. We then used principal component analysis (PCA), as a non-supervised exploratory statistical tool to detect the variables responsible for the greater variance in the CCO activity data due to the age and laser variables. In this analysis, the samples are grouped according to their similarity, without prior information on the groups studied.

Second, we used hypothesis-testing statistical procedures to compare the group mean differences of CCO activity in the specific brain regions (Figure 2) using standard ANCOVA with the whole-brain mean as a covariate. The detailed statistical analysis of the ANCOVA for each region of interest are presented in the Supplementary Table 1. A statistical group difference was considered when the *p*-value was lower than 0.05.

Finally, we used correlative analysis of the functional neuroanatomical systems made of regions showing significant effects to determine systems-level effects in each of the four groups. Correlative analysis measures the association between continuous variables, rather than compare discrete group means. Specifically, correlative models based on CCO activity express how a change in CCO activity in a brain region is associated with an increase or decrease of CCO activity in another region (Puga et al., 2007). Therefore, the obtained correlation matrices can provide an overview of the functional coupling between brain regions in the different control and treatment groups. We calculated one averaged CCO measurement per region per subject. Then we used the averaged regional data from each functional neuroanatomical system to calculate standard Pearson’s bivariate correlations (*r*-values from 1 to 0) to create correlation matrices among the systems for each of the groups. This correlative analysis using CCO activity data has been established previously as a way to estimate the modification of network functional connectivity in the rat brain (Riha et al., 2011; Vélez-Hernández et al., 2014; Auchter et al., 2020). All analyses were performed using Excel and the Statistical Package for the Social Sciences (SPSS Inc., IBM, version 221.0, Chicago, IL, United States).

## Results

To evaluate whether the laser stimulation (with a laser diode of 810 nm wavelength and 100 mW power) can modulate brain metabolism in different stages of life, we mapped CCO activity throughout the brain of young and aged rats submitted to 58 consecutive days of laser treatment or control treatment. We present first the results of the exploratory PCA that analyzed the general data variance characteristics of the two variables (age and laser). Second, we present the results of the ANCOVA hypothesis-testing that detected specific regional significant differences between the groups. Finally, we present the activity correlations between the four functional systems of regions showing significant group differences to evaluate systems-level effects of age and laser treatment.

### General Cytochrome c Oxidase Effects of Laser Treatment in Young and Aged Rats

Principal component analysis revealed two principal components selected by the exploratory analysis that accounted for 63.7% of the data variance. The first component (PC1) accounted for 54.09% of the data variance, which appeared due to differential age and treatment effects. The second component (PC2) accounted for 9.61% of the data variance, which appeared due to similar laser effects. Exploratory inspection of the overall descriptive dataset indicated that the main effect of aging was to reduce CCO activity, while the main effect of laser stimulation was to increase CCO activity in the older rats to levels similar to the younger control rats.

Second, to determine whether laser treatment produced any non-specific effects on whole-brain metabolism, the CCO measurements from all brain regions were averaged for each of the four groups. There was no significant difference on overall brain metabolic CCO activity {age [*F*_(1,20)_ = 1.880; *p* = 0.186], group [*F*_(1,20)_ = 0.001; *p* = 0.977], and interaction age×group [*F*_(1,20)_ = 0.057; *p* = 0.814)]}. Therefore, the ANCOVA used the whole-brain mean of each subject as a covariate to statistically compare specific brain regions of interest among groups.

### Specific Regional Cytochrome c Oxidase Effects of Laser Stimulation in Young and Aged Rats

The detailed group means and standard errors for each specific region of interest are presented in Table 1. The table shows that regions in the young control (YC) group presented higher CCO activity units than in the aged control (AC) group with few exceptions. The specific regional significant effects on mean CCO activity comprised regions belonging to four major functional neuroanatomical systems. Regions showing significant mean group effects were in the sensorimotor system: APar, Ppa, VBL, CPc, and CN3; limbic system: CA1, mt, and AD; auditory system: Aud and MGV; and visual system: A17, A18a, and LP, in rats from young control (YC), young laser (YL), aged control (AC), and aged laser (AL) groups.

**TABLE 1:** Means and standard errors of cytochrome c oxidase activity (μmol of cytochrome c oxidized/min/g of wet tissue weight) of all regions of interest from YC, YL, AC, and AL groups.

Brain region	Abbreviation	YC (*n*)	YL (*n*)	AC (*n*)	AL (*n*)
Anterior olfactory nucleus	AO	425.4 ± 73.5 (7)	350.8 ± 105.0 (5)	337.1 ± 57.5 (6)	305.8 ± 23.9 (6)
Medial frontal cortex	MFr	219.5 ± 23.5 (7)	182.3 ± 30.3 (5)	171.4 ± 17.0 (6)	164.0 ± 17.6 (6)
Sulcal frontal cortex	SFr	285.4 ± 45.0 (7)	199.3 ± 43.9 (5)	192.8 ± 26.9 (6)	180.8 ± 12.9 (6)
Lateral frontal cortex	LFr	294.9 ± 42.4 (7)	198.4 ± 51.7 (5)	165.3 ± 34.8 (6)	201.0 ± 11.2 (6)
Accumbens nucleus	Acb	162.1 ± 16.3 (6)	128.9 ± 45.1 (5)	115.3 ± 15.9 (6)	114.3 ± 14.8 (6)
Anterior cingulate cortex	ACg	240.5 ± 43.6 (6)	210.1 ± 66.2 (5)	177.9 ± 16.6 (6)	226.6 ± 21.7 (6)
Insular cortex	Ins	142.8 ± 14.8 (6)	92.9 ± 25.1 (5)	118.1 ± 11.9 (6)	138.9 ± 15.6 (6)
Lateral septum	LS	231.6 ± 34.8 (6)	202.9 ± 35.1 (5)	175.3 ± 18.9 (6)	205.2 ± 22.5 (6)
Medial septum	MS	167.0 ± 23.2 (6)	129.1 ± 34.5 (5)	113.6 ± 14.2 (6)	131.3 ± 11.5 (6)
Vertical diagonal band	VDB	209.9 ± 27.1 (6)	151.4 ± 54.0 (5)	136.5 ± 12.3 (6)	136.9 ± 14.1 (6)
**Anterior parietal cortex**	*APar	273.7 ± 53.1 (7)	250.1 ± 29.8 (5)	211.3 ± 12.0 (6)	229.3 ± 16.5 (6)
Caudate putamen-rostral	CPr	231.9 ± 21.7 (7)	187.9 ± 34.0 (5)	206.8 ± 16.0 (6)	232.0 ± 17.6 (6)
Claustrum	Cl	136.6 ± 36.0 (7)	145.9 ± 25.6 (5)	120.2 ± 9.6 (6)	158.6 ± 15.9 (6)
Fornix	f	20.5 ± 17.3 (7)	9.4 ± 18.4 (5)	6.8 ± 8.9 (6)	11.7 ± 3.9 (6)
Globus pallidus	Gp	79.5 ± 17.0 (7)	92.9 ± 8.3 (5)	87.6 ± 13.5 (6)	112.4 ± 7.0 (6)
Horizontal diagonal band	HDB	124.6 ± 34.0 (7)	100.4 ± 11.2 (5)	106.1 ± 7.8 (6)	107.5 ± 15.1 (6)
Lateral preoptic area	PoA	141.5 ± 29.4 (7)	132.1 ± 21.5 (5)	143.9 ± 10.1 (6)	164.3 ± 15.4 (6)
Anterior amygdaloid area	AA	151.5 ± 33.1 (7)	107.9 ± 8.2 (5)	158.6 ± 15.9 (6)	141.9 ± 25.0 (6)
**Anterodorsal thalamic nucleus**	*AD	236.3 ± 39.9 (7)	225.1 ± 15.7 (5)	381.5 ± 40.1 (6)	235.0 ± 27.8 (6)
Lateral hypothalamic area	LH	111.7 ± 13.4 (7)	113.8 ± 13.1 (5)	95.1 ± 16.2 (6)	98.3 ± 5.9 (6)
Lateral olfactory tract nucleus	LOT	307.4 ± 103.0 (7)	220.1 ± 38.4 (5)	166.9 ± 13.5 (6)	155.8 ± 36.4 (6)
Paratenial thalamic nucleus	Pt	211.5 ± 32.0 (7)	167.5 ± 37.8 (5)	180.7 ± 10.0 (6)	156.3 ± 18.3 (6)
Reticular thalamic nucleus	Ret	167.5 ± 32.9 (7)	129.9 ± 43.1 (5)	165.1 ± 9.0 (6)	164.5 ± 16.5 (6)
Suprachiasmatic hypothalamic nucleus	SCH	132.1 ± 80.5 (7)	210.6 ± 64.5 (4)	137.6 ± 12.5 (6)	139.8 ± 12.7 (5)
Supraoptic hypothalamic nucleus	SOH	158.9 ± 43.9 (7)	235.1 ± 19.8 (5)	186.0 ± 62.1 (6)	182.6 ± 46.5 (5)
**Caudal caudate-putamen**	*CPc	195.2 ± 34.0 (7)	192.7 ± 33.1 (5)	221.2 ± 11.5 (6)	251.8 ± 23.8 (6)
Dentate gyrus	DG	221.3 ± 47.8 (7)	222.6 ± 39.3 (5)	234.6 ± 21.4 (6)	243.4 ± 26.3 (6)
Lateral habenula	Hb	198.7 ± 25.5 (7)	171.5 ± 17.9 (5)	174.3 ± 16.5 (6)	200.5 ± 17.1 (6)
**Lateral posterior thalamic nucleus**	*LP	213.9 ± 52.3 (7)	159.0 ± 14.1 (5)	163.2 ± 21.8 (6)	184.0 ± 22.1 (6)
**Mammillothalamic tract**	*mt	163.2 ± 28.4 (7)	139.6 ± 5.2 (5)	148.3 ± 12.5 (6)	197.8 ± 16.6 (6)
Perirhinal cortex	Per	139.3 ± 17.4 (7)	141.5 ± 19.7 (5)	159.2 ± 11.0 (6)	170.3 ± 20.1 (6)
Parafascicular thalamic nucleus	Pf	162.4 ± 39.6 (7)	185.7 ± 22.0 (5)	177.9 ± 11.9 (6)	182.1 ± 12.3 (6)
Prepyriform cortex	Pp	124.1 ± 20.8 (7)	107.9 ± 14.6 (5)	120.0 ± 16.0 (6)	114.5 ± 15.1 (6)
**Posterior parietal cortex**	*Ppa	211.5 ± 39.2 (7)	222.2 ± 39.7 (5)	164.9 ± 16.8 (6)	207.8 ± 25.0 (6)
Subthalamic nucleus	Sth	197.7 ± 75.6 (7)	186.4 ± 82.0 (5)	204.2 ± 51.0 (6)	168.4 ± 36.2 (6)
**Ventral basal thalamic nucleus-lateral**	*VBL	193.3 ± 25.2 (7)	144.0 ± 3.9	165.3 ± 13.4 (6)	198.9 ± 12.3 (6)
Ventral basal thalamic nucleus-medial	VBM	208.3 ± 38.3 (7)	175.0 ± 19.3 (5)	180.8 ± 27.3 (6)	208.6 ± 19.6 (6)
Zona incerta	Zi	181.4 ± 24.2 (7)	137.4 ± 17.9 (5)	159.4 ± 27.2 (6)	182.8 ± 21.4 (6)
**Auditory cortex**	*Aud	198.7 ± 8.7 (7)	149.1 ± 6.5 (4)	165.9 ± 13.0 (5)	188.8 ± 19.9 (5)
**Field CA1 of hippocampus**	*CA1	141.7 ± 18.0 (7)	152.8 ± 10.9 (4)	165.9 ± 13.9 (5)	192.1 ± 12.9 (5)
Field CA2 of hippocampus	CA2	200.8 ± 27.7 (7)	202.3 ± 17.5 (4)	192.1 ± 20.0 (5)	221.6 ± 25.0 (5)
Field CA3 of hippocampus	CA3	203.9 ± 36.4 (7)	191.3 ± 11.6 (4)	205.2 ± 16.4 (5)	292.7 ± 30.7 (5)
Medial geniculate nucleus-dorsal	MGD	161.5 ± 19.6 (7)	151.5 ± 10.2 (5)	162.7 ± 20.1 (5)	152.9 ± 16.8 (5)
Medial geniculate nucleus-medial	MGM	203.9 ± 30.0 (7)	179.0 ± 25.7 (5)	178.3 ± 12.4 (5)	175.8 ± 17.6 (5)
**Medial geniculate nucleus-ventral**	*MGV	180.4 ± 27.3 (7)	186.4 ± 35.0 (5)	187.0 ± 25.3 (5)	182.2 ± 22.5 (5)
Presubiculum	Psub	146.5 ± 17.0 (7)	139.6 ± 9.6 (5)	148.0 ± 14.9 (5)	151.2 ± 14.9 (5)
Subiculum	Sub	164.0 ± 21.0 (7)	154.6 ± 12.4 (5)	165.6 ± 13.9 (5)	211.7 ± 22.3 (5)
**Area 17**	*A17	230.2 ± 39.9 (4)	225.4 ± 47.2 (5)	211.3 ± 24.2 (4)	252.0 ± 33.1 (4)
Area 18	A18	230.3 ± 64.6 (4)	190.0 ± 27.0 (5)	189.5 ± 16.7 (4)	212.5 ± 29.5 (4)
**Area 18a**	*A18a	193.8 ± 44.0 (4)	216.4 ± 35.0 (5)	193.4 ± 21.4 (4)	222.2 ± 44.2 (4)
Central gray	CG	233.5 ± 61.0 (4)	175.0 ± 62.9 (5)	156.9 ± 9.7 (4)	167.7 ± 19.1 (4)
**Nucleus of cranial nerve 3**	*CN3	131.4 ± 29.7 (4)	114.1 ± 23.8 (5)	156.9 ± 31.6 (4)	151.9 ± 29.3 (4)
Deep mesencephalic nucleus	DpMe	132.3 ± 31.6 (4)	68.8 ± 26.0 (5)	101.4 ± 15.2 (4)	93.2 ± 20.9 (4)
Interpeduncular nucleus	Ip	303.0 ± 173.1 (4)	242.5 ± 38.4 (5)	249.0 ± 68.3 (4)	159.6 ± 41.5 (4)
Retrosplenial cortex	Rs	225.4 ± 97.3 (4)	206.4 ± 45.0 (5)	219.5 ± 19.3 (4)	266.3 ± 35.0 (4)
Red nucleus	Red	256.7 ± 41.3 (4)	119.1 ± 54.3 (5)	157.8 ± 8.3 (4)	152.9 ± 25.2 (4)
Superior colliculus-deep layers	SCDp	238.4 ± 20.3 (4)	141.7 ± 36.1 (5)	165.1 ± 8.0 (4)	172.1 ± 19.9 (4)
Superior colliculus-superior layers	SCSu	240.9 ± 78.0 (4)	183.9 ± 57.4 (5)	173.1 ± 9.9 (4)	203.1 ± 19.2 (4)
Ventral tegmental area	VTA	92.8 ± 75.5 (4)	51.6 ± 8.8 (5)	73.1 ± 12.9 (4)	91.9 ± 13.6 (4)

*Bold faced indicate regions showing significant group differences at p < 0.05, as described in the text.

**FIGURE 3:**
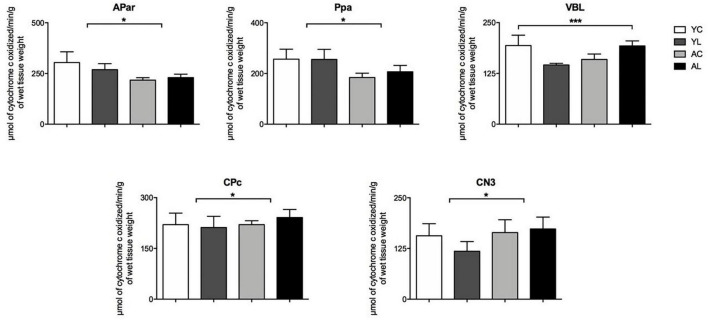
Sensorimotor system. Significant CCO activity group differences in the APar, Ppa, VBL, CPc, and CN3 in rats from young laser (YL; *n* = 5), young control (YC; *n* = 7), aged laser (AL; *n* = 6), and aged control (AC; *n* = 6) groups (*p* < 0.05; ANCOVA). (*) One main effect in the APar, Ppa, CPc, and CN3. (***) Interactions of age and treatment in the VBL. See text for details.

**FIGURE 4:**
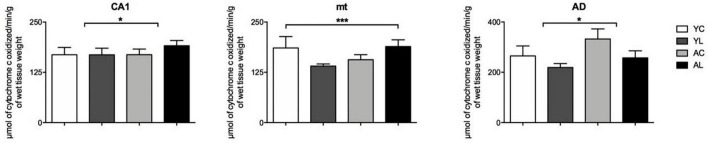
Limbic system. Significant CCO activity group differences in the CA1, mt, and AD in rats from young laser (YL; *n* = 5), young control (YC; *n* = 7), aged laser (AL; *n* = 6) and aged control (AC; *n* = 6) groups (*p* < 0.05; ANCOVA). (*) One main effect in the CA1 and AD. (***) Interactions of age and treatment in the mt. See text for details.

**FIGURE 5:**
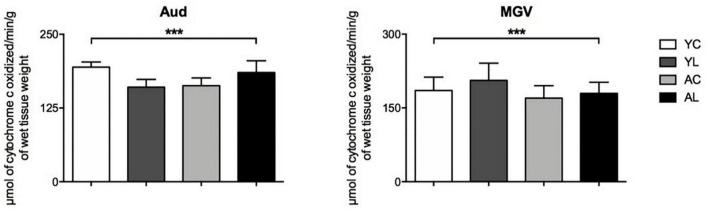
Auditory system. Significant CCO activity group differences in the Aud and MGV in rats from young laser (YL; *n* = 5), young control (YC; *n* = 7), aged laser (AL; *n* = 6) and aged control (AC; *n* = 6) groups (*p* < 0.05; ANCOVA). (***) Interactions of age and treatment in the Aud and MGV. See text for details.

**FIGURE 6:**
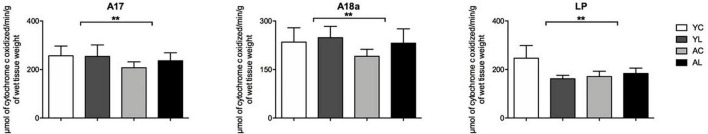
Visual system. Significant CCO activity group differences in the A17, A18a, and LP in rats from young laser (YL; *n* = 5), young control (YC; *n* = 7), aged laser (AL; *n* = 6) and aged control (AC; *n* = 6) groups (*p* < 0.05; ANCOVA). (**) One main effect in the A17, A18a, and LP. See text for details.

(1) Sensorimotor system, which included somatosensory and motor cortex, ventral thalamus, striatum and cranial nerve nuclei (Figure 3). Specifically, significant effects in sensorimotor areas were observed in the APar [*F*_(1,20)_ = 5.189; *p* = 0.034], Ppa [*F*_(1,20)_ = 9.851; *p* = 0.005], VBL [*F*_(1,20)_ = 8.242; *p* = 0.010], CPc [*F*_(1,20)_ = 6.426; *p* = 0.020], and CN3 [*F*_(1,13)_ = 6.171; *p* = 0.029]. The direction of mean effects was different in the cortical and subcortical sensorimotor regions. The direction of the main aging effect was a reduction of CCO in the aged groups in the cortical regions (APar and Ppa). The direction of the main laser effect was a CCO reduction in the younger rats and an increase in the older rats in the subcortical nuclei (VBL, CPc, and CN3). Therefore, younger and older rats showed opposite laser effects on the CCO activity of sensorimotor regions.

(2) Limbic system, which included hippocampus, mammillothalamic tract and anterior thalamus (Figure 4). Specifically, significant mean effects were found in CA1 [*F*_(1,18)_ = 6.360; *p* = 0.022], mt [*F*_(1,20)_ = 4.870; *p* = 0.040], and the AD [*F*_(1,20)_ = 10.839; *p* = 0.004]. The CA1 and mt showed both laser-induced CCO mean increases in the older rats. The AD showed an aging-related mean increase in older rats and a main laser effect of reducing CCO in both younger and older rats. Therefore, in the three limbic regions the direction of the laser effect was opposite to that of the aging effect.

(3) Auditory system, which included the auditory cortex and auditory thalamus (Figure 5). Significant interaction effects of age by treatment group were found in the Aud [*F*_(1,18)_ = 5.162; *p* = 0.036] and MGV [*F*_(1,18)_ = 6.536; *p* = 0.020]. Opposite laser effects were seen in the auditory cortex of younger and older rats. The Aud showed decreased CCO in the younger rats, while it showed increased CCO activity in the older rats to levels similar to the younger control rats.

(4) Visual system, including primary and secondary visual cortex and a visual thalamic nucleus (Figure 6). Specifically, significant mean effects of treatment group were noted in the A17 [*F*_(1,13)_ = 4.914; *p* = 0.047], A18a [*F*_(1,13)_ = 9.853; *p* = 0.009], and LP [*F*_(1,20)_ = 4.790; *p* = 0.041]. The visual cortex regions showed aging-related CCO decreases that were reversed by laser treatment.

### Systems-Level Cytochrome c Oxidase Effects of Age and Laser Stimulation

**FIGURE 7:**
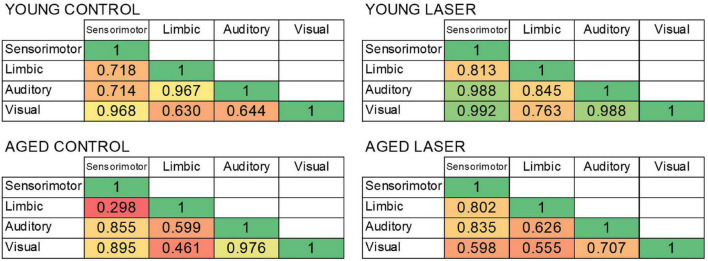
Bivariate correlations between the four functional systems showing significant differences in CCO activity in rats from young laser (YL; *n* = 5), young control (YC; *n* = 7), aged laser (AL; *n* = 6) and aged control (AC; *n* = 6). The heat map shows weaker correlations in red/orange and stronger correlations in yellow/green.

Figure 7 shows the CCO activity correlations (*r*-values) between the four functional neuroanatomical systems with significant regional effects of age and treatment for each of the four groups. The correlations were all positive. Overall, the *r*-values of the aged laser group were more similar to those of the young control group. Specifically, the young control group showed large positive correlation values among the functional systems with *r* ≥ 0.630. The young laser group showed even larger positive correlation values with *r* ≥ 0.763. The aged control group showed the lowest positive correlation values starting with *r* ≥ 0.298. The aged laser group showed positive correlation values larger than the aged control group, starting with *r* ≥ 0.555. Interestingly, all the correlations between the limbic system and the other systems were greater after laser stimulation in the aged treated group as compared to the aged control. In particular, the limbic-sensorimotor correlation value changed the most, from 0.298 in the aged control to 0.802 in the aged laser group.

## Discussion

Although many molecules may be involved in PBM effects, CCO is by far the most abundant photoacceptor in neurons and its absorption peaks have been shown to coincide with the action spectra of PBM *in vitro* (Karu, 1999; Wong-Riley et al., 2005). The available CCO studies in humans using bbNIRS indicate acute effects of PBM on prefrontal cortex CCO oxidation, not chronic effects throughout the brain (Wang et al., 2017a; Saucedo et al., 2021). The present histochemical study is the first to show that transcranial PBM modulates regional CCO activity in multiple systems throughout the brain. The long-time stimulation used here (58 days) is also much longer than used in the other published histochemical study on effects of PBM on CCO activity in the rat brain (Rojas et al., 2012). Our treatment time frame is definitely long enough to induce upregulation or downregulation of the synthesis of new CCO subunits from both mitochondrial and nuclear DNA that would be reflected histochemically as chronic alterations in CCO activity (Gonzalez-Lima and Cada, 1998a).

The most important findings were that aging predominantly decreased brain CCO activity, while chronic laser PBM predominantly reversed the aging effects on brain CCO activity. The PCA exploratory analysis suggested that age and its interaction with treatment accounted for most of the CCO data variance, whereas common treatment effects accounted for about 10% of data variance. Thus, a major effect of chronic laser stimulation in aged brains appeared to be to increase CCO in the laser-treated aged rats to levels more similar to those of the young control rats. Furthermore, the chronic laser effects on brain CCO are consistent with our previous brain metabolomics study, showing that the same chronic laser treatment in aged rats induces brain metabolite values more similar to those of young control rats (Cardoso et al., 2021a).

In contrast, the predominant effect of chronic laser stimulation in young rats appeared to be decreasing brain CCO activity in the laser-treated young rats. The brains of healthy young rats already had high baseline levels of CCO activity. The continued stimulation for 58 consecutive days resulted in a decrease of this baseline activity in many brain regions. The opposite direction of chronic laser effects in young and aged brains resembles the biphasic, hormetic dose-response found more generally for PBM, with lower doses having opposite effects to higher doses (Huang et al., 2011). Therefore, chronic PBM dosimetry for young and aged brains should consider the dose-response phenomenon of hormesis as a PBM dose that may be effective for older brains with low baseline, may be too high for younger brains with high baseline. A hormetic dose-response (also known as U-shaped or bell-shaped dose response) involves stimulation of a biological process at a low dose and inhibition of that process at a high dose (Calabrese, 2008). Huang et al. (2011) published an extensive review showing the hormetic dose-response relationship of PBM. This includes studies done *in vitro*, in animal models, and in humans. For example, Table 1 in Huang et al. (2011) cites 14 *in vitro* studies showing biphasic dose responses, including animal and human cell lines. Table 2 in Huang et al. (2011) cites six studies showing PBM hormesis in animal models such as mice and rats. Therefore, the hormetic response to PBM has been well-documented, and may play a role in the age-related results reported here. It is known that young control rats showed more CCO activity than the aged control rats in most regions, which suggests the possibility that chronic laser for 58 consecutive days yielded opposite outcomes on CCO due to the different baseline levels of oxidative energy metabolism seen in young vs. aged brains. Our previous comparison of acute laser effects in young and old people showed greater CCO effects in the older individuals (Saucedo et al., 2021). This suggests that the aged brain may be more likely to increase CCO after chronic PBM due to its lower baseline levels of CCO, when compared to young brains.

However, the overall brain mean analysis indicated that there was no significant difference on whole-brain metabolic CCO activity between groups, because while some regions showed increases, other regions showed decreases or no changes. The significant effects of aging and laser treatment were more specific to regions of the brain belonging to four major functional systems: sensorimotor, limbic, auditory and visual. It is unlikely that these regional effects of the laser may be due simply to the proximity of brain regions to the locations of the laser probes in the skull, because the laser effects included cortical regions close to the probe (e.g., sensorimotor cortex), as well as much deeper subcortical regions (e.g., CN3 motor nucleus in the midbrain). Thus, it appears that the laser effects were more effective for certain functional neuroanatomical systems than for others.

Further correlative analysis of systems-level CCO activity covariance showed that the laser treatment elevated positive correlations among the modified functional systems, suggesting greater functional connectivity (Puga et al., 2007; Riha et al., 2011). This laser effect was seen in both the treated young and aged groups, and resulted in the aged laser group having systems-level correlative values more similar to those of the young control. An increased systems-level strength in functional coupling after laser treatment has been found previously by PBM using whole-brain mapping of oxygenated hemoglobin changes in the human brain (Urquhart et al., 2020). Together, these systems-level network effects may be interpreted as laser-induced stronger functional connectivity among the stimulated brain systems.

### Limitations and Future Directions

First, the 810 nm wavelength of light used here is not the same 1,064 nm wavelength used in the bbNIRS studies cited earlier that showed PBM of CCO in the human cerebral cortex. To our knowledge, there are no studies demonstrating that an 810 nm wavelength laser can produce PBM of CCO in the human brain. Second, small numbers of subjects per group limited the statistical power of the analysis. Therefore, Bonferroni correction by the large number of regions means that only the largest possible effects would be significant. Even a modified Bonferroni correction is a severe penalty given the large number of regions of interest.

The behavior of the rats was not quantified, because the objective of the experiment was only to quantify brain metabolic changes in CCO activity. However, in a previous study (Cardoso et al., 2021d), the behavior of young and old rats subjected to chronic PBM was investigated, including exploratory activity and habituation in the open field, anxiety in the elevated plus maze, spatial memory in the Barnes maze, and aversive memory in a step-down inhibitory avoidance task. The only difference noted was that young and aged rats submitted to transcranial laser exhibited better cognitive performance in the Barnes maze than did control rats. Future studies will involve additional quantification of behavioral and physiological measures in response to this chronic PBM treatment.

Future directions will include validating the CCO effects induced by chronic PBM in humans. We have been able to monitor CCO changes in response to acute PBM treatments in humans, using broadband near-infrared spectroscopy (Saucedo et al., 2021) in younger and older humans. Future experiments will extend this work to include the study of chronic long-term PBM treatments.

## Conclusion

Regional CCO activity and functional connectivity was decreased in the aged brain, while chronic laser PBM predominantly reversed the aging effects on brain CCO activity. The observed opposite direction of activation effects in treated young and aged brains resembled the biphasic, hormetic dose-response of PBM, indicating that 58 consecutive days of transcranial laser PBM may be too high a dose of stimulation for the young brain, because it downregulated regional CCO activity. However, the strengthening of functional connectivity after laser stimulation was in the same direction in both young and aged groups, although it was stronger in the aged group. These results are consistent with our previous comparison of PBM acute effects on the brains of young and older humans, showing that PBM has a more effective upregulation of CCO in the older brain (Saucedo et al., 2021). Together, these studies suggest that chronic PBM may be an effective treatment to counteract the age-related decline in brain CCO activity and improve oxidative energy metabolism. This PBM effect may help improve the brain and behavioral functions of older individuals with compromised energy metabolism.

## Data Availability Statement

The raw data supporting the conclusions of this article will be made available by the authors, without undue reservation.

## Ethics Statement

The animal study was reviewed and approved by the Ethics Committee, University of Mogi das Cruzes (UMC) (# 004/2020).

## Author Contributions

FG-L and FC designed the experiment, analyzed the data, and wrote the manuscript. DB analyzed the data, prepared the figures, and revised the manuscript. ZW assisted with tissue processing. SG contributed to the experimental design and revision of the manuscript. All authors contributed to the article and approved the submitted version.

## Conflict of Interest

The authors declare that the research was conducted in the absence of any commercial or financial relationships that could be construed as a potential conflict of interest.

## Publisher’s Note

All claims expressed in this article are solely those of the authors and do not necessarily represent those of their affiliated organizations, or those of the publisher, the editors and the reviewers. Any product that may be evaluated in this article, or claim that may be made by its manufacturer, is not guaranteed or endorsed by the publisher.
