# Supplementary material for: Photobiomodulation of Cytochrome c Oxidase by Chronic Transcranial Laser in Young and Aged Brains
Source: Front Neurosci. 2022 Mar 18;16:818005. doi: 10.3389/fnins.2022.818005 (PMC8971717; doi:10.3389/fnins.2022.818005)
Supplement: Supplementary file 1 [file Table_1.docx]

**Supplementary Table 1.** Statistical analysis of CCO activity. ***** Significant difference (p<0.05; ANCOVA).

| Brain region | AGE | GROUP | INTERACTION |
| --- | --- | --- | --- |
| AO | F_(1,20)_=0.002; p=0.964 | F_(1,20)_=0.010; p=0.922 | F_(1,20)_=0.623; p=0.440 |
| MFr | F_(1,20)_=1.054; p=0.318 | F_(1,20)_=0.014; p=0.908 | F_(1,20)_=1.430; p=0.246 |
| SFr | F_(1,20)_=0.015; p=0.903 | F_(1,20)_=0.119; p=0.734 | F_(1,20)_=0.207; p=0.654 |
| LFr | F_(1,20)_=0.342; p=0.565 | F_(1,20)_=0.181; p=0.675 | F_(1,20)_=0.022; p=0.884 |
| Acb | F_(1,19)_=0.094; p=0.762 | F_(1,19)_=0.080; p=0.780 | F_(1,19)_=3.502; p=0.078 |
| ACg | F_(1,19)_=0.033; p=0.857 | F_(1,19)_=1.101; p=0.308 | F_(1,19)_=0.349; p=0.562 |
| Ins | F_(1,19)_=3.836; p=0.066 | F_(1,19)_=0.852; p=0.368 | F_(1,19)_=0.975; p=0.337 |
| LS | F_(1,19)_=1.481; p=0.239 | F_(1,19)_=0.116; p=0.737 | F_(1,19)_=1.216; p=0.285 |
| MS | F_(1,19)_=2.997; p=0.101 | F_(1,19)_=1.905; p=0.184 | F_(1,19)_=0.606; p=0.446 |
| VDB | F_(1,19)_=1.958; p=0.179 | F_(1,19)_=0.279; p=0.604 | F_(1,19)_=0.865; p=0.365 |
| APar | F_(1,20)_=5.189; p=0.034* | F_(1,20)_=0.002; p=0.967 | F_(1,20)_=0.923; p=0.349 |
| CPr | F_(1,20)_=0.086; p=0.773 | F_(1,20)_=0.052; p=0.822 | F_(1,20)_=0.160; p=0.694 |
| Cl | F_(1,20)_=0.150; p=0.702 | F_(1,20)_=0.126; p=0.727 | F_(1,20)_=0.257; p=0.618 |
| Gp | F_(1,20)_=0.951; p=0.342 | F_(1,20)_=0.063; p=0.804 | F_(1,20)_=1.185; p=0.290 |
| HDB | F_(1,20)_=0.182; p=0.674 | F_(1,20)_=0.936; p=0.345 | F_(1,20)_=0.092; p=0.766 |
| PoA | F_(1,20)_=2.715; p=0.116 | F_(1,20)_=0.276; p=0.606 | F_(1,20)_=0.124; p=0.729 |
| AA | F_(1,20)_=1.091; p=0.311 | F_(1,20)_=1.005; p=0.330 | F_(1,20)_=0.530; p=0.477 |
| AD | F_(1,20)_=10.839; p=0.004* | F_(1,20)_=3.834; p=0.067 | F_(1,20)_=0.719; p=0.408 |
| LH | F_(1,20)_=3.149; p=0.094 | F_(1,20)_=0.006; p=0.940 | F_(1,20)_=0.080; p=0.781 |
| LOT | F_(1,20)_=3.593; p=0.075 | F_(1,20)_=0.020; p=0.890 | F_(1,20)_=2.151; p=0.161 |
| Pt | F_(1,20)_=1.581; p=0.226 | F_(1,20)_=0.130; p=0.723 | F_(1,20)_=0.031; p=0.862 |
| Ret | F_(1,20)_<0.0001; p=0.990 | F_(1,20)_=0.006; p=0.938 | F_(1,20)_=0.019; p=0.891 |
| SCH | F_(1,18)_=0.384; p=0.544 | F_(1,18)_=0.419; p=0.526 | F_(1,18)_=0.105; p=0.750 |
| SOH | F_(1,19)_=0.482; p=0.497 | F_(1,19)_=0.305; p=0.588 | F_(1,19)_=0.396; p=0.538 |
| CPc | F_(1,20)_=6.426; p=0.020* | F_(1,20)_=0.707; p=0.411 | F_(1,20)_=0.069; p=0.795 |
| DG | F_(1,20)_=0.723; p=0.406 | F_(1,20)_=0.158; p=0.695 | F_(1,20)_=0.758; p=0.395 |
| Hb | F_(1,20)_=0.072; p=0.792 | F_(1,20)_=0.045; p=0.834 | F_(1,20)_=0.949; p=0.342 |
| LP | F_(1,20)_=0.263; p=0.614 | F_(1,20)_=4.790; p=0.041* | F_(1,20)_=3.087; p=0.095 |
| mt | F_(1,20)_=2.417; p=0.137 | F_(1,20)_=0.090; p=0.767 | F_(1,20)_=4.870; p=0.040* |
| Per | F_(1,20)_=1.058; p=0.317 | F_(1,20)_=1.805; p=0.195 | F_(1,20)_=0.541; p=0.471 |
| Pf | F_(1,20)_=0.071; p=0.793 | F_(1,20)_=0.269; p=0.610 | F_(1,20)_=0.335; p=0.569 |
| Pp | F_(1,20)_=0.039; p=0.846 | F_(1,20)_=0.327; p=0.574 | F_(1,20)_=0.079; p=0.781 |
| Ppa | F_(1,20)_=9.851; p=0.005* | F_(1,20)_=1.213; p=0.285 | F_(1,20)_=0.453; p=0.509 |
| Sth | F_(1,20)_=0.343; p=0.565 | F_(1,20)_=0.065; p=0.802 | F_(1,20)_=2.037; p=0.170 |
| VBL | F_(1,20)_=2.023; p=0.171 | F_(1,20)_=0.236; p=0.633 | F_(1,20)_=8.242; p=0.010* |
| VBM | F_(1,20)_=0.668; p=0.424 | F_(1,20)_=0.274; p=0.607 | F_(1,20)_=0.210; p=0.652 |
| Zi | F_(1,20)_=1.342; p=0.261 | F_(1,20)_=1.168; p=0.293 | F_(1,20)_=0.938; p=0.345 |
| Aud | F_(1,18)_=0.126; p=0.727 | F_(1,18)_=1.039; p=0.322 | F_(1,18)_=5.162; p=0.036* |
| CA1 | F_(1,18)_=6.360; p=0.022* | F_(1,18)_=1.005; p=0.330 | F_(1,18)_=0.021; p=0.888 |
| CA2 | F_(1,18)_=0.043; p=0.838 | F_(1,18)_=2.985; p=0.102 | F_(1,18)_=0.181; p=0.676 |
| CA3 | F_(1,18)_=0.432; p=0.520 | F_(1,18)_=0.113; p=0.740 | F_(1,18)_=1.636; p=0.218 |
| MGD | F_(1,18)_=0.013; p=0.909 | F_(1,18)_=0.895; p=0.357 | F_(1,18)_=0.213; p=0.650 |
| MGM | F_(1,18)_=1.673; p=0.213 | F_(1,18)_=1.645; p=0.217 | F_(1,18)_=0.391; p=0.540 |
| MGV | F_(1,18)_=0.300; p=0.591 | F_(1,18)_=0.765; p=0.394 | F_(1,18)_=6.536; p=0.020* |
| Psub | F_(1,18)_=0.081; p=0.779 | F_(1,18)_=0.023; p=0.881 | F_(1,18)_=0.277; p=0.606 |
| Sub | F_(1,18)_=2.991; p=0.102 | F_(1,18)_=0.473; p=0.501 | F_(1,18)_=0.289; p=0.598 |
| A17 | F_(1,13)_=0.286; p=0.602 | F_(1,13)_=4.914; p=0.047* | F_(1,13)_=0.685; p=0.424 |
| A18 | F_(1,13)_=0.299; p=0.595 | F_(1,13)_=0.081; p=0.780 | F_(1,13)_=0.777; p=0.395 |
| A18a | F_(1,13)_=0.404; p=0.537 | F_(1,13)_=9.853; p=0.009* | F_(1,13)_=0.897; p=0.362 |
| CG | F_(1,13)_=2.162; p=0.167 | F_(1,13)_=0.740; p=0.407 | F_(1,13)_=1.311; p=0.275 |
| CN3 | F_(1,13)_=6.171; p=0.029* | F_(1,13)_<0.0001; p=0.986 | F_(1,13)_=0.082; p=0.779 |
| DpMe | F_(1,13)_=0.020; p=0.890 | F_(1,13)_=1.138; p=0.307 | F_(1,13)_=1.342; p=0.269 |
| Ip | F_(1,13)_=0.025; p=0.877 | F_(1,13)_=2.078; p=0.175 | F_(1,13)_=0.059; p=0.812 |
| Rs | F_(1,13)_=2.686; p=0.127 | F_(1,13)_=2.002; p=0.186 | F_(1,13)_=0.023; p=0.883 |
| Red | F_(1,13)_=1.782; p=0.207 | F_(1,13)_=1.198; p=0.295 | F_(1,13)_=0.018; p=0.895 |
| SCDp | F_(1,13)_=0.549; p=0.473 | F_(1,13)_=1.034; p=0.329 | F_(1,13)_=0.250; p=0.626 |
| SCSu | F_(1,13)_=2.782; p=0.121 | F_(1,13)_=1.647; p=0.224 | F_(1,13)_=0.996; p=0.338 |
| VTA | F_(1,13)_=0.039; p=0.847 | F_(1,13)_=0.269; p=0.614 | F_(1,13)_=1.464; p=0.250 |
